# Survival of trauma patients requiring massive transfusion protocol activation: A retrospective cohort study at a tertiary hospital in Malaysia

**DOI:** 10.1016/j.htct.2026.106489

**Published:** 2026-06-27

**Authors:** Mohd Redzuan Abdullah, Thaenujah Nair Ramachandran, Aidil Noor Adnan, Azian Md Yacob, Siti Fatimah Mohamed Kamaruzzaman, Ahmad Zakimi Abdullah, Nurul Aiman Addenan, Maryam Sumaiya Ahmad Termizi, Joan Gan Cheau Yan, Seniyah Md Sikin, Low Wen Ying, Azarina Zakaria, Wagner Loo Cheng Poh, Zulkifli Hassan, Alex Lim Tat Leong, Siti Aisah Hassan

**Affiliations:** a Transfusion Medicine Unit, Pathology Department, Hospital Sultan Ismail, Ministry of Health Malaysia, Jalan Mutiara Emas Utama, Taman Mount Austin, 81100 Johor Bahru, Johor, Malaysia; b Emergency and Trauma Department, Hospital Sultan Ismail, Ministry of Health Malaysia, Jalan Mutiara Emas Utama, Taman Mount Austin, 81100 Johor Bahru, Johor, Malaysia; c Surgical Department, Hospital Sultan Ismail, Ministry of Health Malaysia, Jalan Mutiara Emas Utama, Taman Mount Austin, 81100 Johor Bahru, Johor, Malaysia; d Anaesthesiology Department, Hospital Sultan Ismail, Ministry of Health Malaysia, Jalan Mutiara Emas Utama, Taman Mount Austin, 81100 Johor Bahru, Johor, Malaysia; e Orthopaedic Department, Hospital Sultan Ismail, Ministry of Health Malaysia, Jalan Mutiara Emas Utama, Taman Mount Austin, 81100 Johor Bahru, Johor, Malaysia; f Radiology Department, Hospital Sultan Ismail, Ministry of Health Malaysia, Jalan Mutiara Emas Utama, Taman Mount Austin, 81100 Johor Bahru, Johor, Malaysia

**Keywords:** Trauma injury, Blood transfusion, Massive transfusion protocol, Glasgow coma scale, Haemoglobin

## Abstract

**Background:**

Trauma is one of the main causes of morbidity and mortality worldwide. Understanding the factors affecting the survivability of trauma patients is crucial, especially those requiring the activation of the massive transfusion protocol. This study aimed to determine survival outcomes and identify factors associated with mortality among trauma patients requiring massive transfusion activation.

**Materials and methods:**

This was a retrospective cohort study of 71 trauma patients who required activation of the massive transfusion protocol. The trauma patients were divided into Survivor (n = 41) and Non-survivor groups (n = 30). The activation criteria for massive transfusions were an assessment of blood consumption score ≥2.

**Results:**

The hospital mortality rate for trauma patients who required activation of the massive transfusion protocol was 42.3%. Most of the patients were male (90.1%), of Malay ethnicity (49.3%), and had blood group O (40.8%). The commonest cause of trauma was road traffic accidents (84.5%). Patients with severe Glasgow Coma Scores had approximately 4.2 times higher odds of mortality compared with those with non-severe Glasgow Coma Scores (p-value = 0.014). Haemoglobin level was independently associated with outcome, with lower haemoglobin levels observed among non-survivors (adjusted odds ratio: 1.38; 95% CI: 1.03–1.85; p-value = 0.030).

**Conclusions:**

The survivability of trauma patients who need massive transfusion protocol activation was associated with Glasgow Coma Score and haemoglobin level on arrival. It is crucial to determine factors that affect the survivability of trauma patients for the early identification of those at risk and to initiate prompt management and resuscitation in the future.

## Introduction

Trauma is one of the leading causes of morbidity and mortality worldwide. In 2021, injuries in Southeast Asia affected an estimated 35.5 million people and caused approximately 317,000 deaths, highlighting a substantial regional mortality burden [1]. Haemorrhage is the most common preventable cause of death for trauma patients [2,3]. The risk of hospital mortality is increased by uncontrolled haemorrhage, which leads to a lethal triad vicious cycle of coagulopathy, hypothermia, and acidosis [4]. Effective coordination between the trauma team is pivotal to ensure the timely management of massively haemorrhage trauma patients.

Implementing the massive transfusion protocol (MTP) is crucial in replacing blood loss and maintaining haemodynamics until definitive care is provided. It is a proactive, standardised protocol designed to facilitate communication between different services and avoid delays in management, including blood product transfusion, for patients requiring a massive transfusion [5]. Early delivery of blood products through predefined MTP in resuscitation was associated with decreased length of hospital stay and mortality rate [6,7]. Trauma patients presenting with low systolic blood pressure, high heart rate, low haemoglobin (Hb), elevated international normalised ratio (INR), decreased base excess, and high lactate have a significantly higher risk of requiring massive transfusion [8].

Mortality risk factors of trauma patients found in previous studies were age, comorbidity, mechanism of injury, Glasgow Coma Scale (GCS), pulse rate, respiratory rate, blood pressure, cardiopulmonary resuscitation performance, blood transfusion, and occurrence of complications [9,10]. Currently, there is limited local and regional data evaluating survival outcomes and mortality-associated factors among trauma patients requiring MTP activation in Southeast Asia. Our hospital functions as a referral trauma centre; however, outcome performance and determinants of mortality have never been evaluated. Therefore, this study was conducted to determine survival outcomes and identify factors associated with mortality among trauma patients requiring MTP activation. This study provides context-specific evidence and actionable predictors to enhance protocol implementation in middle-income country trauma systems.

## Materials and methods

### Research setting and ethics

This was a retrospective cohort study that included all trauma patients who required activation of the MTP (n = 71) at Hospital Sultan Ismail Johor Bahru (HSIJB) from 1^st^ June 2022 to 31^st^ May 2024. The activation criteria for MTP were an Assessment of Blood Consumption (ABC) score ≥2. As the protocol was newly implemented, a simple trigger was selected to ensure timely activation and workflow compliance that will be evaluated as local outcome data accumulate. HSIJB is a specialist tertiary hospital with 704 beds located in Johor Bahru, Johor, Malaysia. It is a leading East Johor hospital cluster and receives referrals from the district hospitals. Ethical approval for this study was obtained from the Medical Research and Ethics Committee, Ministry of Health Malaysia (research number NMRR ID-24-02690-RL4).

### Massive transfusion protocol for trauma patients

An institutional MTP for trauma patients was implemented on 1^st^ June 2022. All providers and staff were systematically educated about the protocol before implementation. It is a standardised protocol designed to facilitate communication between trauma team members. Trauma team members were representatives from the Emergency and Trauma Department (ETD), Surgical Department, Anaesthesiology Department, Orthopaedic Department, Transfusion Medicine Unit (TMU), and Radiology Department.

The emergency physician, surgeon, and anaesthesiologist were the authorised personnel to activate the MTP. If the patient was at the ETD, the Surgical Department or ETD medical officer is nominated as the MTP coordinator. The Anaesthesiology Department medical officer was nominated as the MTP coordinator if the patient was in the operation theatre or the Intensive Care Unit (ICU). The coordinator was responsible for notifying the activation or termination of MTP, calling the TMU for box preparation, ensuring cold chain maintenance of blood products, and ensuring compliance with the transfusion procedure, including complete documentation. Upon activation, the coordinator conveys only essential information: the patient’s name, diagnosis, location, ABC score, MTP activator, and the coordinator’s name and contact number.

To initiate the protocol, the first MTP box was requested by sending a completed transfusion request form and 6 mL of the patient’s whole blood to the TMU. The sample was collected in a 5.4 mg K2​EDTA BD Vacutainer (Becton, Dickinson and Company, Franklin Lakes, NJ, USA). For the subsequent MTP box, a second blood transfusion request form and an additional 3 mL whole blood sample were required.

**Fig. 1:**
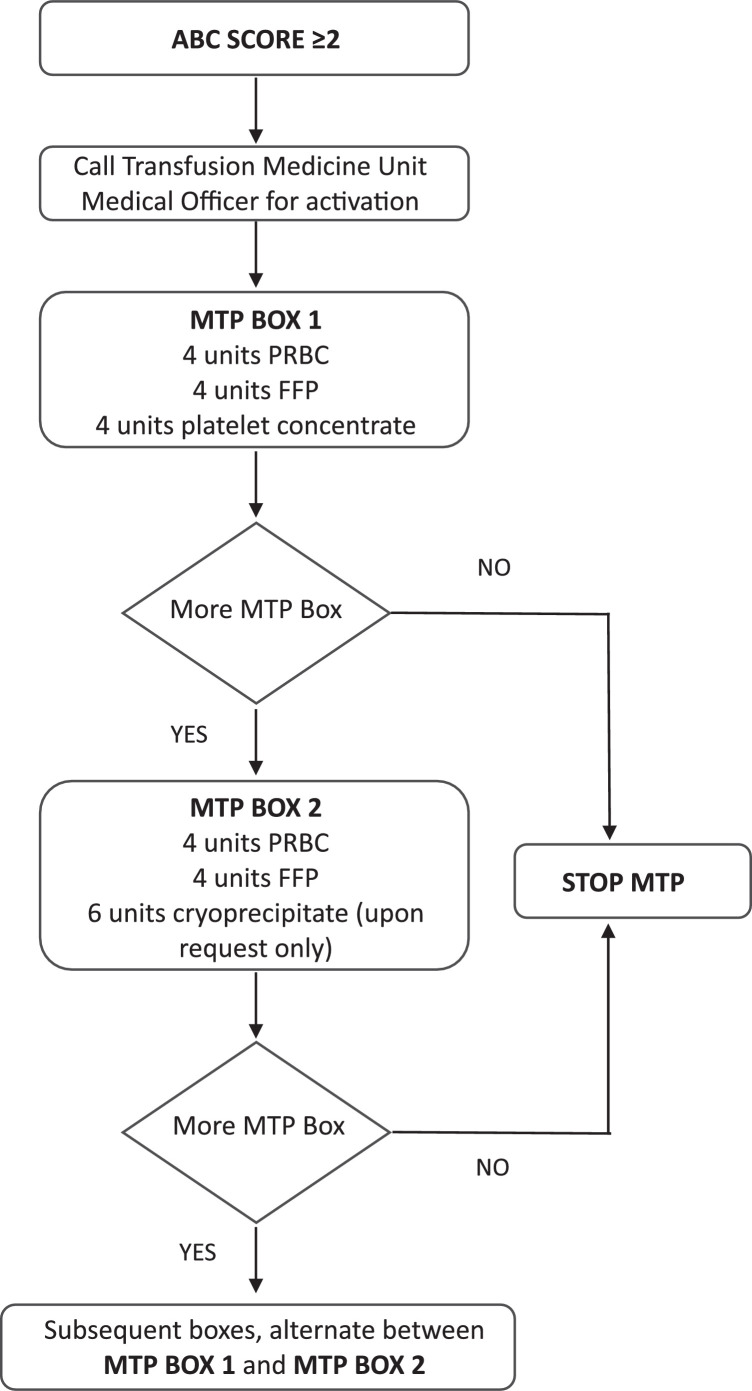
Massive transfusion protocol for trauma.

The standard MTP Box 1 contained four units of group-crossmatched (GXM) packed red blood cells (PRBCs), four units of fresh frozen plasma (FFP), and four units of platelet concentrate. To maintain a 1:1 PRBC-to-FFP ratio, the number of GXM PRBC units was reduced by the number of "Safe O" units (Group O Rh(D) positive, direct Coombs test negative) already supplied during the emergency phase. The standard MTP box 2 contained four fully crossmatched PRBCs and four units of FFP. In addition, six units of cryoprecipitate were added to box 2 upon request by the attending clinician based on clinical assessment and fibrinogen level (if available). Subsequent MTP boxes were alternated between box 1 and box 2 if MTP needed to be continued (Figure 1). For transportation, an insulated blood box with an ice pack was used for PRBC or FFP, and without ice for platelets or cryoprecipitate. Screening for transfusion-transmitted infections in all blood and blood components was negative. The laboratory turnaround time for each box was 30 minutes.

Whenever possible, patients or their authorised next-of-kin signed written informed consent forms for blood transfusions. However, two fully registered clinicians decided on urgent blood transfusions if no family member was available. The patients were transfused according to ABO-specific blood components. A haematology analyser XN-450, Sysmex Corporation, Japan, was used for point-of-care testing of the complete blood count (CBC). A viscoelastic haemostatic assay test was not available at the hospital.

If the patient was in the ETD, the attending emergency physician or surgeon was responsible for MTP deactivation and the attending anaesthesiologist or surgeon was responsible for MTP deactivation if the patient was in the operating theatre or ICU. The MTP deactivation criteria included both anatomic (control of bleeding) and physiological (haemodynamically stable) statuses.

### Variables and research tools

Patient data were collected regarding demographics (age, gender, ethnicity, blood group, referral status, comorbidities, and mechanism of trauma) and clinical characteristics (Glasgow Coma Scale [GCS] score, pulse rate, systolic and diastolic blood pressure, and total MTP boxes delivered). Additionally, laboratory parameters (Hb, white blood cell count, and platelet count) and clinical outcomes (adverse transfusion reactions, length of ICU and hospital stay, and mortality) were recorded. All the data was retrieved retrospectively using the Total Hospital Information System (THIS), an institutional electronic medical record platform used for comprehensive clinical documentation, including patient management records and blood bank transfusion data. Survivability was defined as the ability to remain alive and be discharged from the hospital after being admitted due to a traumatic injury.

### Statistical analysis

All the data were tabulated and analysed using the Statistical Package for the Social Science (SPSS) Statistics Version 29 (IBM, New York, USA). Numerical variables were expressed as median and interquartile range (IQR) and categorical variables were expressed as count and percentage. Simple logistic regression was performed to examine associations between clinical and laboratory variables and survival, followed by multiple logistic regression to identify factors independently associated with survival. Results are presented as adjusted odds ratios (OR) with 95% confidence intervals (95% CIs). The level of significance was set at a p-value <0.05.

## Result

### Demographics of the patients

The hospital mortality rate for trauma patients who required activation of the MTP was 42.3%. The cohort was predominantly male, reflecting the typical distribution of severe trauma in our setting. Road traffic accidents accounted for the majority of cases, indicating that high-energy blunt trauma was the principal mechanism leading to MTP activation (Table 1). Other mechanisms, including falls, industrial injuries, and assault, were relatively uncommon. The ABO blood group was unknown for two patients due to early mortality after MTP was activated.

**Table 1:** Demographics of the patients.

Variable	All patients (n = 71)	Non-survivor (n = 30)	Survivor (n = 41)
Age (years) - median (IQR)	33.00 (29)	34.00 (31)	32.00 (28)
Gender – n (%)			
Male	64 (90.1)	28 (93.3)	36 (87.8)
Female	7 (9.9)	2 (6.7)	5 (12.2)
Ethnicity – n (%)			
Malay	35 (49.3)	15 (50.0)	20 (48.8)
Chinese	16 (22.5)	7 (23.3)	9 (22.0)
Indian	7 (9.9)	3 (10.0)	4 (9.8)
Other ethnicities	13 (18.3)	5 (16.7)	8 (19.5)
Blood group – n (%)			
O	29 (40.8)	14 (46.7)	15 (36.6)
A	15 (21.1)	7 (23.3)	8 (19.5)
B	23 (32.4)	6 (20.0)	17 (41.5)
AB	2 (2.8)	1 (3.3)	1 (2.4)
Unknown	2 (2.8)	2 (6.7)	0 (0.0)
Referral case – n (%)			
No	60 (84.5)	27 (90.0)	33 (80.5)
Yes	11 (15.5)	3 (10.0)	8 (19.5)
Comorbidity – n (%)			
No	56 (78.9)	25 (83.3)	31 (75.6)
Yes	15 (21.1)	5 (16.7)	10 (24.4)
Type of comorbidity – n (%)			
Diabetes mellitus	6 (8.5)	2 (6.7)	4 (9.8)
Hypertension	6 (8.5)	2 (6.7)	4 (9.8)
Dyslipidaemia	2 (2.8)	0 (0.0)	2 (4.9)
Cardiovascular disease	4 (5.6)	3 (10.0)	1 (2.4)
Cerebrovascular disease	1 (1.4)	0 (0.0)	1 (2.4)
Respiratory disease	3 (4.2)	2 (6.7)	1 (2.4)
Renal disease	2 (2.8)	2 (6.7)	0 (0.0)
Cause of trauma – n (%)			
Road traffic accident	60 (84.5)	26 (86.7)	34 (82.9)
Industrial injury	2 (2.8)	1 (3.3)	1 (2.4)
Fall	8 (11.3)	3 (10.0)	5 (12.2)
Assault	1 (1.4)	0 (0.0)	1 (2.4)

IQR: Interquartile range

### Clinical characteristics and laboratory parameters of the patients

In most cases, the TMU delivered one (45.1% of patients) or two (36.6% of patients) MTP boxes. The highest number of MTP boxes delivered to patients was five boxes indicated for three patients (4.2%). Lower initial GCS scores and haemoglobin (Hb) levels were observed in the non-survivor group, indicating that these patients presented with greater physiological compromise than survivors (Table 2).

**Table 2:** Clinical characteristics and laboratory parameters of the patients.

Variable	All patients (n = 71)	Non-survivor (n = 30)	Survivor (n = 41)
GCS on arrival – n (%)			
Severe (score ≤ 8)	24 (33.8)	16 (53.3)	8 (19.5)
Non-severe (score > 8)	47 (66.2)	14 (46.7)	33 (80.5)
Pulse rate on arrival (beats/minute) – median (IQR)	120.00 (33)	120.00 (24)	122.00 (38)
Systolic blood pressure on arrival (mmHg) – median (IQR)	92.00 (44)	90.00 (53)	93.00 (41)
Diastolic blood pressure on arrival (mmHg) – median (IQR)	61.50 (31)	62.00 (39)	61.00 (29)
Haemoglobin on arrival (g/dl) – median (IQR)	13.00 (2.4)	12.40 (4.5)	13.10 (1.4)
White cell count on arrival (x10^3^/µL) – median (IQR)	19.00 (10.3)	18.90 (8.7)	19.10 (11.6)
Platelet count on arrival (x10^3^/µL) – median (IQR)	244.00 (73)	234.00 (61)	251.00 (81)

GCS: Glasgow Coma Scale; IQR: Interquartile range

### Outcome of the patients

The median length of ICU stay was five days, while the median total hospital stay was 12 days (Table 3) reflecting the substantial resource utilisation of trauma patients requiring MTP activation. Transfusion-related adverse events were uncommon with only two cases of mild allergic reactions and no severe transfusion reactions being reported. Both cases were limited to urticaria and resolved with standard treatment.

**Table 3:** Outcome of the patients.

Variable	All patients (n = 71)	Non-survivor (n = 30)	Survivor (n = 41)
Adverse transfusion reaction – n (%)			
No	69 (97.2)	30 (100.0)	39 (95.1)
Yes	2 (2.8)	0 (0.0)	2 (4.9)
Length of ICU stay (days) – median (IQR)	5.00 (11)	1.00 (4)	8.00 (7)
Length of hospital stay (days) – median (IQR)	12.00 (22)	2.00 (7)	17.00 (21)
Time of death – n (%)			
≤ 6 hours	NA	7 (23.3)	NA
6 < x ≤ 24 hours		7 (23.3)	
> 24 hours		16 (53.3)	

ICU: Intensive Care Unit; IQR: Interquartile range; NA: Not Applicable

### Independent factors associated with non-survival

Patients with severe GCS had approximately 4.2 times higher odds of mortality compared with those with non-severe GCS (p-value = 0.014). Additionally, Hb level was independently associated with outcome, with lower Hb levels observed among non-survivors (adjusted OR: 1.38; 95% CI: 1.03–1.85; p-value = 0.030) (Table 4).

**Table 4:** Variable logistic regression modelling

Variable	Simple logistic regression		Multiple logistic regression	
	Crude OR (95% CI)	p-value	Adjusted OR (95% CI)	p-value
GCS on arrival				
Severe (score ≤8)	0.21 (0.07-0.61)	0.004	0.24 (0.08-0.75)	0.014
Non-severe (score >8)	1	NA	1	NA
Pulse rate on arrival	1.00 (0.98-1.02)	0.878	NA	NA
Systolic blood pressure on arrival	1.00 (0.98-1.02)	0.914	NA	NA
Diastolic blood pressure on arrival	1.00 (0.98-1.02)	0.893	NA	NA
Haemoglobin on arrival	1.37 (1.05-1.79)	0.021	1.38 (1.03-1.85)	0.030
White blood cell count on arrival	0.99 (0.94-1.05)	0.815	NA	NA
Platelet level on arrival	1.00 (1.00-1.01)	0.372	NA	NA

GCS: Glasgow Coma Scale; OR: Odds ratio; 95% CI: 95% Confidence interval; NA: Not applicable

P-value <0.05 is considered significant

## Discussion

The mortality rate of trauma patients who needed MTP activation ranged from 16.9% to 67.0% [[11], [12], [13], [14], [15]]. Based on the results of this study, the majority of trauma patients who needed MTP activation were male with road traffic accidents being the most common mechanism of injury. These findings were consistent with the results of other studies [13,16,17]. Males were more frequently involved in road traffic accidents, which may be attributed to a higher prevalence of high-risk behaviors, such as speeding, driving under the influence (DUI), and aggressive driving [18]. The National Trauma Database of eight public hospitals in Malaysia showed males were 6.5 times more likely to be involved in major trauma than females. The majority of cases resulted from blunt trauma (96.3%) and were unintentional in nature (91.2%), with road traffic accidents (76.8%) being the most frequent cause of injury [19].

Delays in MTP activation and delivery of blood components were associated with prolonged duration to achieve haemostasis and reduced chances of survival [20]. Massive blood transfusion has been defined in various articles as the replacement of an entire blood volume within 24 hours, transfusion of more than ten units of PRBC in 24 hours, transfusion of more than four units of PRBC within one hour with expectation for more transfusion support, or replacement of 50% of total blood volume within three hours [[21], [22], [23]]. The temporal-based definition is not useful during the acute management of severe haemorrhage trauma patients. Therefore, a simpler and reliable predictive score is needed that helps clinicians activate MTP faster. The ABC score had a sensitivity between 60% and 89% and a specificity between 84% and 86% [[24], [25], [26]]. The advantage of the ABC scoring system lies in its use of non-laboratory variables, those derived from history, physical examination, and Focused Assessment with Sonography in Trauma (FAST) scans. These parameters are easily applied in ETD settings and facilitate early resource mobilisation.

The observed association between low GCS scores and mortality is clinically plausible, as a markedly reduced neurological status may reflect either primary traumatic brain injury or cerebral hypoperfusion secondary to haemorrhagic shock. In patients requiring MTP activation, the GCS likely represents a surrogate marker of overall injury burden and physiological compromise at presentation. The trauma patients who required massive transfusions had a lower GCS score, higher pulse rate, lower systolic blood pressure, lower Hb level, higher Injury Severity Score (ISS), higher INR, and longer hospital stay [27]. The GCS is widely used to assess the level of consciousness of trauma patients at the site of injury and in the hospital. Previous studies found that the GCS score was a predictive factor for mortality after trauma [28,29]. A retrospective study involving 513 trauma patients found that a unit increment of the GCS score resulted in a 40% decrease in the risk of death (OR: 0.63; 95% CI: 0.59–0.67; p-value <0.05) [16].

Identifying trauma patients with severe bleeding on hospital arrival is crucial to initiate appropriate intervention and life-saving procedures such as massive transfusion and damage control resuscitation. In the initial part of acute bleeding, the Hb and haematocrit levels may appear normal because red blood cells and plasma are lost concomitantly [30]. Lower Hb level in trauma patients often reflects the severity of haemorrhage and ongoing blood loss, which can lead to reduced oxygen delivery to vital organs and subsequent organ dysfunction. The decreases in the Hb level on hospital arrival are associated with trauma severity and the requirement for haemostasis intervention [31]. Bruns et al. reported that a Hb ≤10 g/dL was associated with significant haemorrhage in 87% of trauma patients and a three-fold increase in the need for interventions to stop the haemorrhage (OR: 3.14; 95% CI: 1.18-8.35; p-value <0.05) [32]. In addition, severe haemorrhage may lead to trauma-induced coagulopathy and tissue hypoperfusion, which further worsens patient outcomes. In this setting, early activation of MTP is essential to facilitate rapid and balanced blood component replacement, thereby restoring circulating volume, improving oxygen delivery, and correcting coagulopathy. Calcium and fibrinogen levels are currently not routinely monitored within our institution’s massive transfusion protocol, highlighting an opportunity to enhance practice following evolving clinical guidelines.

The length of hospital stay in this study (median: 12 days) was consistent with the average length of hospital stays for major trauma patients in other hospitals in Malaysia (ranging from 3-18 days) [19]. Among trauma patients, factors affecting the length of hospital stay are age, gender, mechanism of injury, infection, type of injury, and Injury Severity Score (ISS) [33]. This study found that 46.6% of trauma patients who needed MTP activation succumbed within 24 hours of admission. A retrospective analysis of 1,470 major trauma patients found that 68% of deaths occurred within the first 48 hours and were mainly due to haemorrhagic shock and traumatic brain injury [34].

This study provides institution-specific data on outcomes among trauma patients requiring MTP activation. The implementation of MTP facilitates coordinated communication between the trauma team members. However, challenges in implementation remain, including the timeliness of activation and blood product delivery, as well as adherence to the protocol in emergency settings. Higher levels of protocol compliance have been associated with lower mortality, highlighting the importance of timely activation, appropriate laboratory monitoring, and protocol-based administration of blood products [35]. Although this study did not directly evaluate protocol adherence or transfusion timing, the findings provide preliminary institutional data that may support ongoing evaluation and optimisation of the MTP workflow. Measures to improve compliance may include periodic education of healthcare providers, discussion of MTP cases during trauma team meetings, regular MTP simulation drills, and placement of clear protocol flowcharts in key areas such as the trauma bay and transfusion medicine unit.

This study has several limitations, including its retrospective design, single-centre setting, and relatively small sample size. In addition, adjustment for potential confounders was limited due to incomplete data. Important variables such as injury severity scoring, transfusion volume, timing, and biochemical markers, including lactate and international normalised ratio (INR), were not consistently available in the dataset.

## Conclusion

In conclusion, the survivability of trauma patients who need MTP activation was associated with the GCS score and Hb level on arrival at the hospital. It is crucial to determine factors that affect the survivability of trauma patients for early identification of those at risk and to initiate prompt management and resuscitation in the future.

## Funding

This research received no specific grant from funding agencies in the public, commercial, or not-for-profit sectors.

## Data availability statement

The data that support the findings of this study are available from the corresponding author upon reasonable request.

## Conflicts of interest

The authors declare no conflicts of interest.
